# Synthesis, Crystal Structure, and Compressibilities of Mn_3−*x*_Ir_5_B_2+*x*_ (0≤*x*≤0.5) and Mn_2_IrB_2_

**DOI:** 10.1002/chem.201803235

**Published:** 2018-09-20

**Authors:** Benedikt Petermüller, Christopher Neun, Michal Stekiel, Dominik Zimmer, Martina Tribus, Klaus Wurst, Björn Winkler, Hubert Huppertz

**Affiliations:** ^1^ Institut für Allgemeine, Anorganische und Theoretische Chemie Leopold-Franzens-Universität Innsbruck Innrain 80-82 A-6020 Innsbruck Austria; ^2^ Institut für Geowissenschaften, Abteilung für Kristallographie Goethe-Universität Frankfurt am Main Altenhöferallee 1 D-60438 Frankfurt Germany; ^3^ Institut für Mineralogie und Petrographie Leopold-Franzens-Universität Innsbruck Innrain 52 A-6020 Innsbruck Austria

**Keywords:** borides, compressibility, crystal structures, density functional calculations, EDX measurements, high-temperature synthesis

## Abstract

The new ternary transition metal borides Mn_3‐*x*_Ir_5_B_2+*x*_ (0≤*x*≤0.5) and Mn_2_IrB_2_ were synthesized from the elements under high temperature and high‐pressure/high‐temperature conditions. Both phases can be synthesized as powder samples in a radio‐frequency furnace in argon atmosphere. High‐pressure/high‐temperature conditions were used to grow single‐crystals. The phases represent the first ternary compounds within the system Mn–Ir–B. Mn_3−*x*_Ir_5_B_2+*x*_ (0≤*x*≤0.5) crystallizes in the Ti_3_Co_5_B_2_ structure type (*P*4/*mbm*; no. 127) with parameters *a*=9.332(1), *c*=2.896(2) Å, and *Z*=2. Mn_2_IrB_2_ crystallizes in the β‐Cr_2_IrB_2_ crystal structure type (*Cmcm*; no. 63) with parameters *a*=3.135(3), *b*=9.859(5), *c*=13.220(3) Å, and *Z*=8. The compositions of both compounds were confirmed by EDX measurements and the compressibility was determined experimentally for Mn_3−*x*_Ir_5_B_2+*x*_ and by DFT calculations for Mn_2_IrB_2_.

## Introduction

Metal borides display a variety of very interesting physical properties such as a high hardness (ReB_2_, WB_4_, FeB_4_, IrB_1.35_),^1^ low compressibility (OsB_2_, Re_7_B_3_),^2^ magnetic properties (Nd_2_Fe_14_B),^3^ high transition temperature into a superconducting state (MgB_2_),^4^ and a very high electron emissivity (LaB_6_).^5^ Most borides can be synthesized at ambient pressure and are therefore relatively inexpensive and easily accessible, which makes them interesting materials for industrial usage.1a,1b, 6 Very few compounds exhibit such a huge structural variety as borides do, which can be seen from the existence of more than 1000 binary and ternary borides that crystallize in over 150 different structure types.^7^ The discovery of superconducting MgB_2_ below 39 K in 2001 by Nagamatsu et al.^4^ led to a tremendous interest in borides within the scientific community. Even before the discovery of the superconductivity of MgB_2_, some borides such as Ru_7_B_3_ and Mo_2_IrB_2_ were known to be superconducting at very low temperatures.^8^ The compound Mo_2_IrB_2_ was first synthesized and characterized by Rogl et al. in 1972 and the superconductivity was detected a few years later by Vandendberg et al.8a, 9 Since then only few other borides, such as Cr_2_IrB_2_ and Mo_2_OsB_2_, were found to crystallize in the same crystal structure.^10^ Kotzott et al. revisited the crystal structure of Cr_2_IrB_2_ in 2007 and were successful in synthesizing β‐Cr_2_IrB_2_, which crystallizes in a structure type similar to that of Mo_2_IrB_2_.^11^

Here, we report the synthesis of Mn_2_IrB_2_, representing the first ternary Mn–Ir–B compound and the second known phase adopting the β‐Cr_2_IrB_2_ structure type. We furthermore synthesized Mn_3−*x*_Ir_5_B_2+*x*_ (0≤*x*≤0.5), a second phase within the system Mn–Ir–B that crystallizes in the Ti_3_Co_5_B_2_ structure type. The Ti_3_Co_5_B_2_ structure type (in general *A*_3_ *T*_5_B_2_), which was first described by Kuz′ma et al. in 1971, and including related structures, such as the quaternary substitution variant with the general formula of *A*_2_ *MT*_5_B_2_, is one of the most common structure types within metal‐rich borides.^12^ In the quaternary variant, the “Ti” position is split into two different crystallographic sites with different coordinations: a pentagonal‐prismatic “A”‐position and a tetragonal‐prismatic coordinated “M”‐position. Within the quaternary variant, the atoms occupying the “M”‐position are usually smaller than those occupying the “A”‐position. The “T”‐position is preferentially occupied by a valence electron‐rich transition metal such as Co, Rh, or Ir. The occupation of the “M”‐position with a magnetically active element (e.g. Cr, Mn, Fe, Co, Ni) leads to compounds with notable magnetic properties such as ferromagnetism in Sc_2_MnIr_5_B_2_, anti‐ferromagnetism in Sc_2_FeIr_5_B_2_, or meta‐magnetism in Sc_2_MnRh_5_B_2_.^13^ Due to the huge variety of possible elements occupying the various positions, over 60 compounds are known to adopt the crystal structure of the aristotype or the quaternary and quinary variants, respectively.^12^, ^13^, ^14^ Despite its importance, up to now only eight ternary compounds crystallizing in the Ti_3_Co_5_B_2_ type are known.^15^ With the successful synthesis of Mn_3−*x*_Ir_5_B_2+*x*_ (0≤*x*≤0.5), a new ternary member with a magnetic active element (Mn) at the important “M” position can be added to the important family of compounds crystallizing in the Ti_3_Co_5_B_2_ structure type.

## Results and Discussion

### Crystal structure of Mn_2_IrB_2_

**Figure 1:**
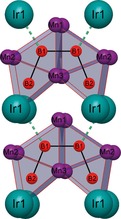
Two B_4_ units with the BM_6_ prisms.

**Figure 2:**
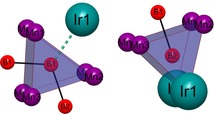
Two different BM_6_ prism. Left: B1Mn_6_ prism capped by two boron and one iridium atom. Right: B2[Mn_4_Ir_2_] capped by one B1 atom.

**Figure 3:**
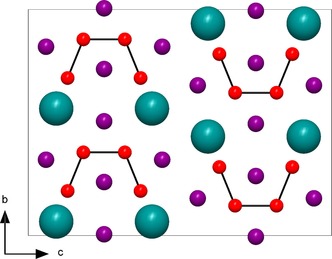
Unit cell of Mn_2_IrB_2_ with the BM_6_ units aligned in two different orientations. Boron atoms in red, manganese atoms in purple, and iridium atoms displayed in cyan. An illustration of the anisotropic displacement ellipsoids can be found in the Supporting Information (Figure S3).

Mn_2_IrB_2_ is the first known ternary phase within the system Mn–Ir–B. According to the systematic extinctions, the orthorhombic, centrosymmetric space group *Cmcm* was derived for Mn_2_IrB_2_. The dimensions of the unit cell are *a*=3.135(3), *b*=9.859(5), *c*=13.220(3) Å, and *V*=408.57(4) Å^3^ with *Z*=8 formula units. The compound is isotypic to β‐Cr_2_IrB_2_ 11 with Mn occupying the Cr positions. The refinement with free occupancy factors showed that one crystallographic site (Mn3) is not entirely occupied by manganese atoms, but depicts a partial substitution of ≈7(2) % of the manganese atoms by iridium atoms. As the atomic radii of iridium (1.35 Å) and manganese (1.40 Å) are similar, such a partial substitution can be expected.^16^ The boron atoms form a B_4_ chain that can be interpreted as a fragment of a hexagon (Figure 1). The chain consists of two different boron atoms with an interatomic distance of 1.808(2) Å for B1−B1 and 1.813(9) Å for B1−B2. The B2‐B1‐B1 angle within the chain amounts to 112.2(3)° which is relatively close to the ideal 120° angle within a hexagon. Six metal atoms in the form of a trigonal BM_6_ prism (Figure 2) coordinate the boron atoms, whereas there are two different kinds of BM_6_ prisms. Six Mn atoms build up the trigonal prisms, which coordinate the two B1 atoms in the center of the chain, whereas four Mn and two Ir atoms form the two prisms, which coordinate the B2 atoms at the end of the chain fragments (Figure 1 and 2). The B1−Mn distance within the B1Mn_6_ prisms range from 2.227(4)–2.283(5) Å, being capped by the two neighboring B atoms (B1 and B2) and by one Ir atom (B1−Ir1: 2.244(6) Å) (Figure 1). The interatomic distances within the B2M_6_ prisms range from 2.264(5)–2.283(5) Å for B2−Mn and 2.213(4) Å for B2−Ir. The complete building unit can also be described as four trigonal BM_6_ prisms interconnected by their rectangular sides arranged in a “*cis*” geometry. The unit cell contains four of these BM_6_ units, where two are orientated with the “open” side of the chain fragment upwards and the other two units downwards (Figure 3).

The β‐Cr_2_IrB_2_ structure type is closely related to the well‐known Mo_2_IrB_2_‐type. The main difference is the arrangement of the B_4_ chain fragment. Whereas in the β‐Cr_2_IrB_2_ type the chain can be seen as a fragment of a hexagon (Figure 1), in the Mo_2_IrB_2_ type the B_4_ unit represents a fragment of a zig‐zag boron chain. The interatomic distances between the atoms in Mn_2_IrB_2_ and in β‐Cr_2_IrB_2_ are very similar, which was expected as the only difference is the substitution from the chromium atoms by manganese atoms both being of similar size.^9^, ^11^

### Crystal structure of Mn_3−*x*_Ir_5_B_2+*x*_ (0≤*x*≤0.5)

**Figure 4:**
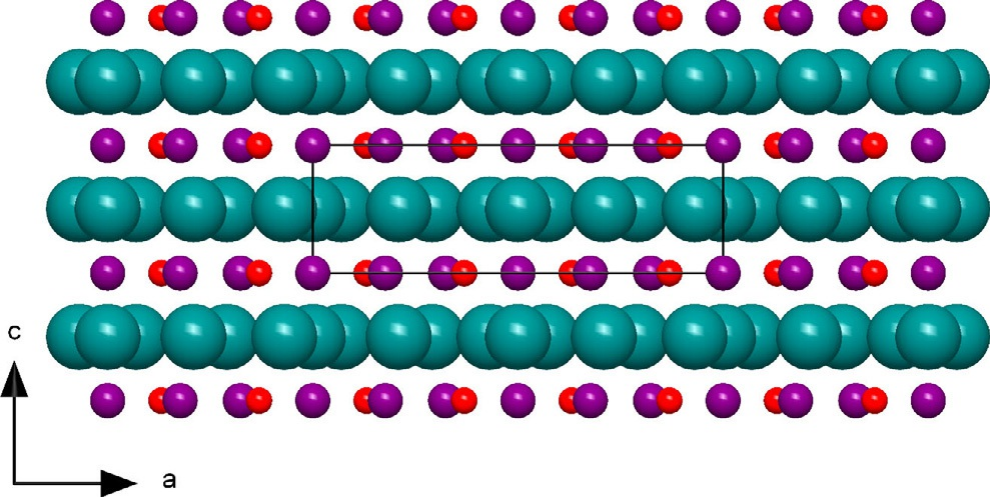
Layered structure of Mn_3−*x*_Ir_5_B_2+*x*_ (0≤*x*≤0.5) with alternating layers of iridium (cyan) or manganese (purple) and boron (red) atoms. An illustration of the anisotropic displacement ellipsoids can be found in the Supporting Information (Figure S4).

**Figure 5:**
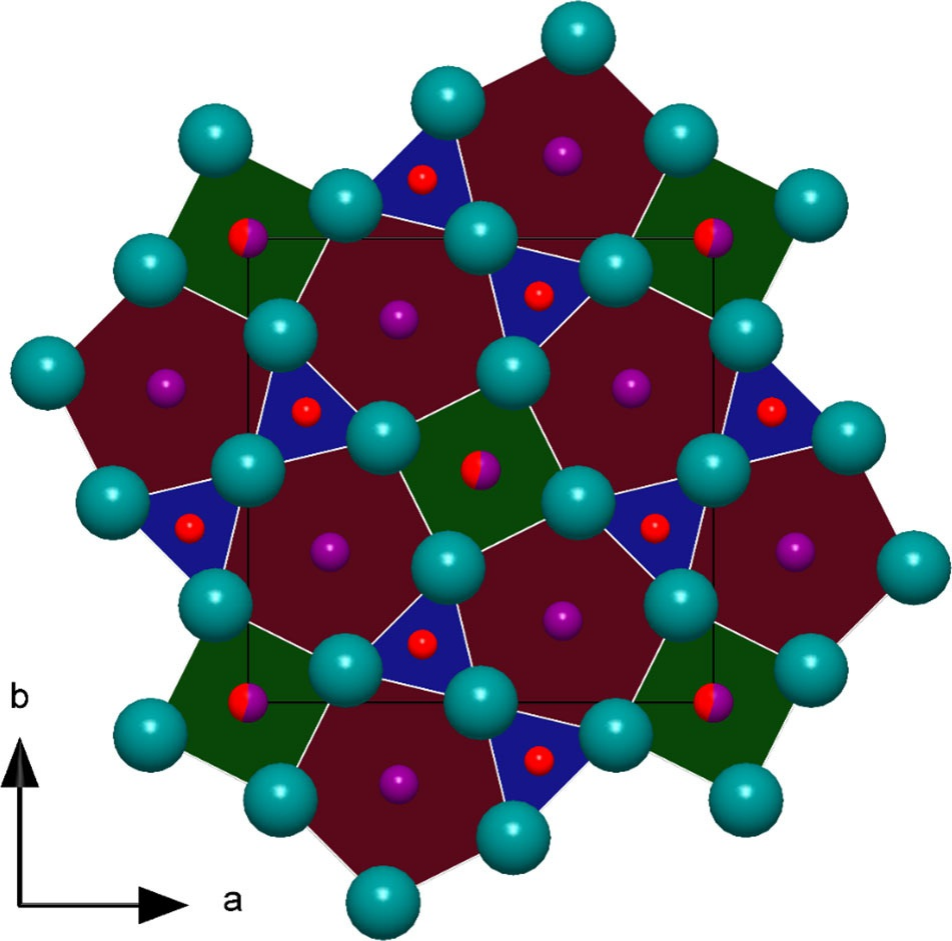
Different columns formed by the iridium atoms (cyan) along [0 0 1]. Edge‐sharing, trigonal prisms (blue) centered by boron atoms (red). Manganese atoms (purple) are located within pentagonal (bordeaux red) channels and the Mn/B mixed site in the center of the tetragonal (green) channels.

**Figure 6:**
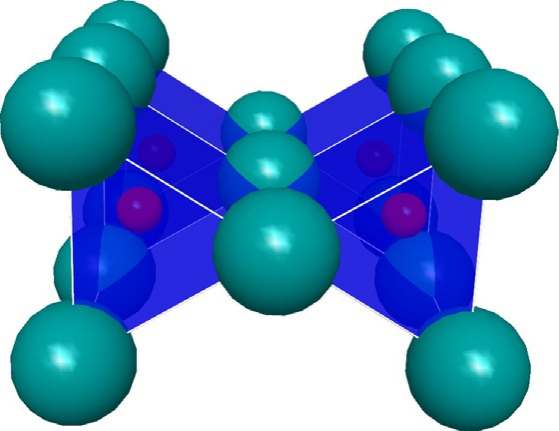
Column of face‐ and edge‐sharing trigonal BIr_6_ prisms.

The new compound Mn_3−*x*_Ir_5_B_2+*x*_ (0≤*x*≤0.5) represents the second known ternary phase within the system Mn–Ir–B. From the systematic extinctions, the tetragonal space group *P*4/*mbm* (no. 127) was derived. The dimensions of the unit cell are *a*=9.332(1) and *c*=2.896(2) Å with *V*=252.19(2) Å^3^. Mn_3−*x*_Ir_5_B_2+*x*_ (0≤*x*≤0.5) is isostructural to Ti_3_Co_5_B_2_, which was first described by Kuz′ma et al. in 1971.12a The structure determination revealed that the Wyckoff‐position 2*a* is a subject of substitutional disorder between Mn (55(5) %) and B (45(5) %) atoms. In order to indicate the phase width, the phase is labelled as Mn_3−*x*_Ir_5_B_2+*x*_ (0≤*x*≤0.5). The structure is built up by alternating layers (ABAB) consisting either of iridium atoms or of manganese and boron atoms (Figure 4). By stacking the iridium layers in the *c*‐direction, columns of face‐sharing trigonal, tetragonal, and pentagonal iridium polyhedra are formed (Figure 5). The boron and manganese atoms reside within these different channels. The trigonal iridium prisms are centered by the boron atoms with the boron‐iridium distance ranging from 2.16(1) to 2.18(1) Å. The BIr_6_ prisms within one column share their rectangular faces with pentagonal MnIr_10_ polyhedra of the neighboring channel and furthermore one common Ir2–Ir2 edge with another column of BIr_6_ prisms, leading to a column of edge‐sharing trigonal prisms (Figure 6). Eight iridium atoms form a cuboid with edge lengths of 2.882(1) and 2.900(1) Å and coordinate the Mn/B atoms at the 2*a* site with an Ir−Mn/B distance of 2.506(1) Å. Further manganese atoms (Mn2) reside within the center of pentagonal iridium prisms with a side length ranging from 2.773(1)–2.900(1) Å and angles ranging from 108.1(2)–116.9(1)° for the base face of the pentagonal prism. Three of the rectangular faces are shared with the trigonal BIr_6_ prisms and two with the neighboring tetragonal MnIr_8_ cuboid.

Due to the mixed Mn/B site at the Wyckoff position 2*a*, Mn and B atoms occupy the “Ti” position, whereas the Wyckoff position 4*g* is exclusively occupied by Mn atoms. The different occupation of the two “Ti‐positions” is well established in compounds of the Ti_3_Co_5_B_2_ structure‐type family such as in Ti_2.4(2)_Co_5.6(2)_B_2_ or Sc_2_MnIr_5_B_2_.^13^, 15b The substitution of the metal atom at the 2*a* site by much smaller boron atoms was first observed by Fokwa et al. in Ti_3−*x*_Ru_5−*y*_Ir_*y*_B_2+*x*_ (0≤*x*≤1 and 1≤*y*≤3).^17^ The interaction between the magnetically active atoms (manganese) on this position is decisive for the excellent magnetic properties of the isostructural compounds.^13^, 14c,14f, 15b

### Elemental analysis

Numerous crystals for both phases were investigated with the focus on the Mn:Ir ratio, as the detection of boron is impossible. Mn_2_IrB_2_ shows a ratio of 67.9±0.9 atom % Mn and 32.1±0.9 atom % Ir and for Mn_3−*x*_Ir_5_B_2+*x*_ (0≤*x*≤0.5) a ratio of 35.1±1.8 atom % Mn and 64.9±1.8 atom % Ir was observed. Both ratios are similar to the ratios obtained by the single‐crystal structure determination. As the metal ratios of both phases differ by few atomic percent compared to the single‐crystal structure solution, the exact formula should be Mn_2±*x*_Ir_±x_B_2_ (*x* ≤0.1) and Mn_3−*x*_Ir_5_B_2+*x*_ (0≤*x*≤0.5) to indicate the phase width.

### Compressibility

**Figure 7:**
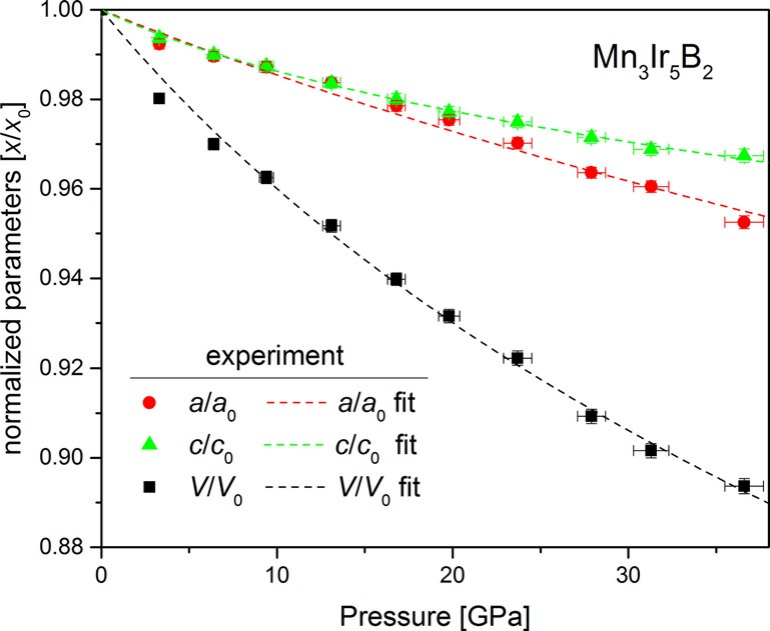
Compression behavior of the lattice parameters of Mn_3‐*x*_Ir_5_B_2+*x*_ (0≤*x*≤0.5) up to 37 GPa. The data were fitted using a third order BM EOS (dashed lines).

**Figure 8:**
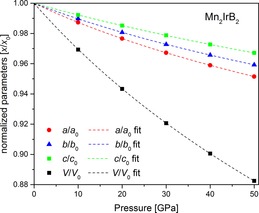
Compression behavior of the unit cell parameters up 50 GPa obtained by DFT‐calculations. The data was fitted using a third order BM EOS (dashed lines).

In the pressure regime up to 37 GPa, Mn_3−*x*_Ir_5_B_2+*x*_ (0≤*x*≤0.5) shows no structural phase transition. The bulk modulus is *B*_0_ (Mn_3−*x*_Ir_5_B_2+*x*_ (0≤*x*≤0.5), 3rd order)=209(10) GPa, with a pressure derivative *B*′=7.8(1.8). Restraining *B*′ to the value 4 gives *B*_0_ (Mn_3−*x*_Ir_5_B_2+*x*_ (0≤*x*≤0.5), 2nd order)=246(6) GPa. The individual lattice parameters show anisotropic behavior as the pressure increases, shown by the *c* lattice parameter becoming more incompressible, which is reasonable regarding the fact that the atomic layers are stacked in the *c*‐direction (Figure 7). A summary of the compressibilities of the phases is given in Table 1, and the pressure dependence of the unit cell parameters is listed in Table S5 (Supporting Information). Due to metrological difficulties, the compressibility of Mn_2_IrB_2_ could not be obtained experimentally but could be determined from DFT calculations (Figure 8).

**Table 1:** Experimental and calculated results of the compressibilities. The DFT values were obtained from stress‐strain relations.

Compound	Space group	BM order	*B*_0_ [GPa] (exp)	*B*′
Mn_3−*x*_Ir_5_B_2+*x*_ (0≤*x*≤0.5)	*P*4/*mbm*	2nd 3rd	246(6) 209(10)	4 7.8(1.8)
				
			*B_0_* [GPa] (DFT)	
Mn_2_IrB_2_	*Cmcm*	2nd 3rd	309(2) 304(2)	4 4.4(2)

### DFT calculations

DFT calculations were performed on a fully ordered structure of Mn_2_IrB_2_, neglecting the partial substitution of Mn atoms by Ir atoms. The pressure dependence of the lattice parameters was calculated up to a maximum pressure of 50 GPa. Fitting the DFT data by a third order Birch–Murnaghan (BM EOS) yielded *B*_0_ (Mn_2_IrB_2_, 3rd order, DFT)=304(2) GPa with a pressure derivative *B*′=4.4(2). A second order fit gives *B*_0_ (Mn_2_IrB_2_, 2nd order, DFT)=309(2). The calculations showed no indications of a structural change upon compression. The anisotropic behavior of the individual lattice parameters remained unchanged over the entire pressure, with *a* being most compressible (Figure 8). A summary of the compressibilities of the phases is given in Table 1 and the pressure dependence of the unit cell parameters is listed in Table S6.

## Conclusions

With the synthesis of Mn_2_IrB_2_ and Mn_3−*x*_Ir_5_B_2+*x*_ (0≤*x*≤0.5), we have synthesized the first two ternary borides containing manganese and iridium. Both phases can be synthesized by high‐temperature techniques but high‐temperature/high‐pressure conditions were used to improve the quality of the single crystals. Mn_2_IrB_2_ represents the second phase crystallizing within the β‐Cr_2_IrB_2_ structure type, whereas Mn_3−*x*_Ir_5_B_2+*x*_ (0≤*x*≤0.5) crystallizes in the well‐known Ti_3_Co_5_B_2_ type, with a Mn/B mixed site. Within the new compound, the important “M”‐position, which is responsible for many outstanding magnetic properties of others phases with the Ti_3_Co_5_B_2_ type, is occupied with a magnetically active element (Mn). Due to the extremely low scattering cross section of boron in comparison with iridium and manganese for X‐rays, it is practically impossible to reliably determine the exact occupation of the boron atoms solely based on X‐ray diffraction data. Therefore, EDX measurements were carried out to specify the existing phase width. Compared to related binary iridium and manganese borides such as β‐Ir_4_B_5_ (*B*_0_, 3rd order, =249(3) GPa), Ir_5_B_4_ (*B*_0_, 3rd order, =304(6) GPa) and MnB_4_ (*B*_0_, 3rd order, =254(9) GPa), Mn_3−*x*_Ir_5_B_2+*x*_ (0≤*x*≤0.5) shows a higher compressibility (*B*_0_, 3rd order, =209(10) GPa). The DFT calculations for Mn_2_IrB_2_ indicate that its compressibility (*B*_0_, 3rd order, DFT=304(2) GPa) is lower than the compressibility of MnB_4_ and comparable to iridium borides, whereby it needs to be confirmed by experiment.^18^ Both bulk moduli are in between the bulk modulus for elemental iridium (*B*_0_, 3rd order, =326(3) GPa) and elemental manganese (120 GPa) but do not reach the high bulk moduli of the rhenium or osmium borides.^2^, ^19^ The existence of a new, manganese‐containing phase crystallizing in the Ti_3_Co_5_B_2_ structure type should stimulate further research, especially focusing on the magnetic properties of the new compounds.

## Experimental Section

### Synthesis

Manganese (99.95 % purity, ChemPur, Karlsruhe, Germany), iridium (99.9+ % purity, ChemPur, Karlsruhe, Germany) and amorphous boron (95+ % purity, Goodfellow, Cambridge, England) were used as starting materials in a molar ratio of 2:1:2 for Mn_2_IrB_2_ and of 1:2:2 for Mn_3−*x*_Ir_5_B_2+*x*_ (0≤*x*≤0.5). The reaction mixtures were finely ground in an agate mortar and afterwards inserted into crucibles made from hexagonal boron nitride (HeBoSint P100, Henze BNP GmbH, Kempten, Germany). The boron nitride crucibles were placed in tungsten crucibles (Plansee Metall GmbH, Reutte, Austria) and heated in a radio frequency furnace (TruHeat HF 5010, Hüttinger Elektronik GmbH+CO. KG, Freiburg, Germany) under Ar atmosphere.^20^ For the synthesis of Mn_2_IrB_2_, the furnace was first heated to 1200 °C within one hour, maintained at 1200 °C for four hours, then the temperature was lowered to 1000 °C within 10 hours, and finally quenched to room temperature by switching off the furnace. The synthesis of Mn_3_Ir_5_B_2_ required a temperature of 1400 °C, which was then held for 12 hours before switching off the furnace. Both compounds were obtained as off blackish powders, but no single‐crystals with an adequate quality and size for single‐crystal X‐ray diffraction analyses could be synthesized.

With the aim to improve the crystal size and quality, high‐pressure/high‐temperature syntheses were carried out. Therefore, the finely grounded educts were inserted in a hexagonal boron nitride (HeBoSint P100, Henze BNP GmbH, Kempten, Germany) container, which was then inserted into a 14/8 high‐pressure assembly. It was compressed to 10 GPa within four hours by a high‐pressure device consisting of a hydraulic 1000 t press (mavo press LPR 1000‐400/50, Max Voggenreiter GmbH, Mainleus, Germany) and a Walker‐type module (Max Voggenreiter GmbH) with eight tungsten carbide cubes (HA‐7 %Co, Hawedia, Marklkofen, Germany). The mixture was heated from ambient temperature to 1100 °C within 5 min. The temperature was held for 45 minutes and afterwards reduced to 700 °C in 45 minutes. For the syntheses of both phases, the same synthesis program can be used. After decompression, the samples were isolated from the surrounding assembly parts by mechanical separation. This method led to the formation of single‐crystals of Mn_2_IrB_2_ and Mn_3−*x*_Ir_5_B_2+*x*_ (0≤*x*≤0.5) with dimensions of up to 0.040 mm and 0.015 mm, respectively. A more detailed description of this setup can be found in the literature.^21^

### High‐pressure X‐ray diffraction

For high‐pressure experiments, Boehler–Almax type diamond anvil cells were used.^22^ The cells were loaded with Ne as pressure‐transmitting medium. Samples were placed in holes of 110–130 μm in diameter, which were drilled by a custom‐built laser lathe in pre‐indented Re gaskets (40–50 μm in thickness). The pressure was determined using the ruby fluorescence method.^23^ Synchrotron X‐ray diffraction experiments at high pressures were performed at the beamline P02.2 of the PETRA III synchrotron (DESY, Hamburg, Germany). The diffraction patterns were acquired with a PerkinElmer XRD1621 detector at a wavelength of 0.2902 Å, with beams focused to 1.5×2.3 μm FWHM by Kirkpatrick–Baez mirrors. A CeO_2_ standard and an Enstatite single‐crystal standard were used to determine the sample‐to‐detector distance and for detector calibration during the experiments.^24^ The diffraction patterns were corrected and integrated using the FIT2D and DIOPTAS software packages.^25^

### Crystal structure analysis

The powder X‐ray diffraction patterns were obtained in transmission geometry from flat samples of the products. The measurements were carried out using a STOE STADI P powder diffractometer equipped with Mo${}_{K\alpha {}_{1}}$ radiation (Ge(111) monochromator *λ*=0.7093 Å) in the 2*θ* range of 2.0–60.3° with a step size of 0.015° for both phases. As a detector, a silicon microstrip solid‐state detector (Dectris Mythen 1 K) was employed. For Mn_3−*x*_Ir_5_B_2+*x*_ (0≤*x*≤0.5), a Rietveld analysis was carried out, which is shown in Figure S1 in Supporting Information. Most of the reflections can be assigned to Mn_3−*x*_Ir_5_B_2+*x*_ (0≤*x*≤0.5), however, a few reflections (e.g. at 18.3°, 20.3°, and 21.5°) could not be assigned to any known phase. Next to the non‐assignable reflections and those of Mn_3−*x*_Ir_5_B_2+*x*_ (0≤*x*≤0.5), the pattern exhibits a broad amorphous halo at low 2*θ* that is due to unreacted amorphous boron. The reflection of the (1 1 0) lattice plane at 6.6° cannot be detected as it is too weak compared to the amorphous halo in this range. The lattice parameters of Mn_3−*x*_Ir_5_B_2+*x*_ (0≤*x*≤0.5) shown in Table 2 were obtained through a Rietveld analysis of the powder pattern using the program TOPAS.^26^

**Table 2:** Crystal data and structure refinement of Mn_2.55(5)_Ir_5_B_2.45(5)_ (standard deviations in parentheses).

Empirical formula	Mn_2.55_Ir_5_B_2.45_
Molar mass [g mol^−1^]	1147.44
Crystal system	tetragonal
Space group	*P*4/*mbm* (no. 127)
**Powder data**	
Powder diffractometer	STOE Stadi P
Radiation	Mo${}_{K\alpha {}_{1}}$ (*λ*=0.7093 Å)
*a* [Å]	9.332(1)
*c* [Å]	2.896(2)
*V* [Å^3^]	252.19(2)
**Single‐crystal data**	
Single‐crystal diffractometer	Esperanto‐Crys/Alis/Pro
Radiation	Synchrotron (*λ*=0.291 Å)
*a* [Å]	9.2850(2)
*c* [Å]	2.8823(5)
*V* [Å^3^]	248.48(5)
Formula units per cell *Z*	2
Calculated density [g cm^−3^]	15.0699
Crystal size [mm]	0.006×0.006×0.015
Temperature [K]	293(2)
Absorption coefficient [mm^−1^]	13.671
*F*(000)	920
*θ* range, deg.	1.80–16.18
Range in *hkl*	−13≤*h*≤14, −15≤*k*≤15, −2≤*l*≤2
Total no. of reflections	529
Independent reflections	192
Reflections with *I*≥2*σ*(*I*)	177
Data/ parameters	192/ 11
Absorption correction	multi‐scan
Goodness‐of‐fit on *F* ^2^	4.04
Final *R* indices [*I*≥2*σ*(*I*)]	*R*1=0.0501
	*wR*2=0.0609
Final *R* indices (all data)	*R*1=0.0501
	*wR*2=0.0609
Largest diff. peak and hole [e Å^−3^]	6.15/−5.80

The powder pattern of Mn_2_IrB_2_ exhibits further reflections, which cannot be assigned to any known phase within the system Mn–Ir–B. These reflections show that at least one further unknown compound was synthesized as a side‐product during this synthesis. Due to the amount of non‐assignable reflections, a Rietveld refinement was unsuccessful and the measured powder pattern of Mn_2_IrB_2_ was just visually compared to the theoretical pattern (Figure S2).

Small single crystals of Mn_2_IrB_2_ were selected by mechanical fragmentation using a polarization microscope. A Bruker D8 Quest Kappa diffractometer with Mo_Kα_ radiation (*λ*=0. 71073 Å) was used to collect the single‐crystal intensity data at room temperature. A multiscan absorption correction (SADABS‐2014)^27^ was applied to the intensity data sets. The structure solution and parameter refinement (full matrix‐least‐squares against *F* ^2^) were performed by using the SHELX‐13 software suite with anisotropic displacement parameters for all atoms.^28^ According to the systematic extinctions, the orthorhombic space group *Cmcm* (no. 63) was derived for Mn_2_IrB_2_. The GOF of 1.235 as well as the value of *R*1=0.0250 for 640 unique reflections with *I*≥2*σ*(*I*) are indicative of a successful refinement. To ensure that no symmetry operations were missing, the final solution was checked with PLATON.^29^ All relevant details of the data collection and the refinement are listed in Table 3, the positional parameters are listed in Table S1 (Supporting Information), and the important bond lengths are listed in Table S2. The program Diamond^30^ was used for the graphical representation (Figure 1–6) of both structures.

**Table 3:** Crystal data and structure refinement of Mn_2_IrB_2_ (standard deviations in parentheses).

Empirical formula	Mn_2_IrB_2_
Molar mass [g mol^−1^]	323.70
Crystal system	orthorhombic
Space group	*Cmcm* (no. 63)
**Single‐crystal data**	
Single‐crystal diffractometer	Bruker D8 Quest Kappa
Radiation	Mo_Kα_ (*λ*=0.71073 Å)
*a* [Å]	3.135(3)
*b* [Å]	9.859(5)
*c* [Å]	13.220(3)
*V* [Å^3^]	408.57(4)
Formula units per cell *Z*	8
Calculated density [g cm^−3^]	10.525
Crystal size [mm]	0.035×0.040×0.025
Temperature [K]	292(2)
Absorption coefficient [mm^−1^]	76.663
*F*(000)	1096
*θ* range, deg.	3.08–37.91
Range in *hkl*	−5≤*h*≤5, −16≤*k*≤16, −22≤*l*≤22
Total no. of reflections	13383
Independent reflections	645
Reflections with *I*≥2*σ*(*I*)	640
Data/ parameters	645/35
Absorption correction	multi‐scan
Goodness‐of‐fit on *F* ^2^	1.235
Final *R* indices [*I*≥2*σ*(*I*)]	*R*1=0.0250
	*wR*2=0.0632
Final *R* indices (all data)	*R*1=0.0252
	*wR*2=0.0633
Largest diff. peak and hole [e Å^−3^]	2.67/−6.43

For Mn_3−*x*_Ir_5_B_2+*x*_ (0≤*x*≤0.5), single‐crystal diffraction data were collected using crystals placed in diamond anvil cells. The data were collected by performing *ω*‐scans in 0.5° steps, with an integration time of 1 second. A platinum foil of proper thickness was used as an absorber in order not to oversaturate a majority of reflections. The CrysAlis program was used to reduce and integrate the data. A standard enstatite crystal was measured beforehand, in order to determine the instrumental parameters. The space group determination and solution of the crystal structure were done with SHELXT,28b the crystal structure refinement was done with Jana2006.^31^ The crystal structure solution and refinement was performed in space group *P*4/*mbm*. Upon crystal structure solution, the positions of Mn and Ir atoms were found. The SHELXT algorithms were not able to determine the positions of the much lighter B atoms; these were taken from DFT calculations. The formula of the resulting compound was Mn_3_Ir_5_B_2_ and resultant structure was found to be isostructural to Ti_3_Co_5_B_2_.^32^ No observed reflections violated the extinctions of the space group *P*4/*mbm*. The correctness of the crystal structure solution was furthermore checked by carrying out a Rietveld refinement of the measured powder diffraction pattern. It clearly emphasizes the assumption of *P*4/*mbm* being the correct space group (Figure S2). Additionally, the DFT calculations also suggest that the structure description in the space group *P*4/*mbm* is the correct one. The structure refinement in the space group *P*4/*mbm* based on the single‐crystal data showed that the isotropic displacement parameter of the Mn1 atom was four times larger than that of the Mn2 atom. This suggested that the model with the formula Mn_3_Ir_5_B_2_ overestimates the electron density at the Mn1 site. In the next step, the data was refined with a partial substitution of the Mn1 site with B atoms. The final refinement showed that such a model fits very well to the data, further lowering the factor *R*_obs_ and yielding reasonable atomic displacement parameters. The final formula was found to be Mn_3−*x*_Ir_5_B_2+*x*_ with *x*=0.45(5). This model was again confirmed by the Rietveld refinement of the powder diffraction data.

All relevant details of the data collection and the refinement are listed in Table 2, the positional parameters are listed in Table S3, and the important bond lengths are listed in Table S4. The program Diamond^30^ was used for the graphical representation (Figures 1–6) of both structures.

Further details of the crystal structure investigation(s) may be obtained from Fachinformationszentrum Karlsruhe, 76344 Eggenstein‐Leopoldshafen, Germany (fax: (+49)7247‐808‐666; e‐mail: crysdata@fiz‐karlsruhe.de, https://www.fiz-Karlsruhe.de/en/leistungen/kristallographie/kristallstrukturdepot/order-form-request-for-deposited-data.html) on quoting the deposition number CSD‐434720 for Mn_2_IrB_2_ and CSD‐434721 for Mn_3−*x*_Ir_5_B_2+*x*_ (0≤*x*≤0.5).

### Elemental analysis

Crystals of both phases were semi‐quantitatively investigated by the use of a JEOL JSM‐6010LV scanning electron microscope with a Quantax (Bruker, Billerica, USA) energy dispersive system (EDX) for element identification. An acceleration voltage of 15 kV was used for small fragments of the sample, which were mounted on carbon pads on a charge reducing sample holder. Suitable regions of the crystals were selected for the measurement points.

### Determination of the compressibility

The compressibilities were determined by investigating the dependence of the unit cell parameters upon compression. The unit cell parameters were obtained by analyzing the single‐crystal data. The data were fitted using a third order Birch–Murnaghan (BM) equation of state^33^ using the EosFit software package.^34^

In Equation (1), *p* is the pressure, *V*_0_ is the reference volume at ambient conditions, *V* is the unit cell volume at the respective pressure and *B*_0_ is the bulk modulus. In the second order BM equation of state, $B_{0}^{{}^{'}}$ is constrained to 4.

(1) $$p\left(V\right)=\frac{3B_{0}}{2}\left[{\left(\frac{V_{0}}{V}\right)}^{\frac{7}{3}}-{\left(\frac{V_{0}}{V}\right)}^{\frac{5}{3}}\right]\left[1+\frac{3}{4}\left(B_{0}^{{}^{'}}-4\right)\left({\left(\frac{V_{0}}{V}\right)}^{\frac{2}{3}}-1\right)\right]\mathrm{with}\;\;B_{0}^{{}^{'}}={\left(\frac{\partial B}{\partial p}\right)}_{p=0}$$

### Density functional theory

In order to obtain a better understanding of the structure‐property relations of the synthesized compounds, we performed density functional theory (DFT) calculations employing the CASTEP^35^ code. This code implements the Kohn–Sham DFT based on a plane wave basis set in conjunction with pseudopotentials. The plane wave basis set is unbiased (as it is not atom‐centered) and does not suffer from basis set superposition errors in comparison to atom‐centered analogues. It also makes converged results straightforward to obtain in practice, as the convergence is controlled by a single adjustable parameter, the plane wave cut‐off, which was set to 580 eV. All pseudopotentials were ultrasoft and were generated using the PBE‐GGA to allow for a fully consistent treatment of the core and valence electrons.^36^ Brillouin zone integrals were performed by using Monkhorst–Pack grids with spacings of less than 0.020 A^−1^ between individual grid points. A simultaneous optimization of the unit cell parameters and internal coordinates was performed in the way that forces were converged to ≤0.005 eV A^−1^ and the stress residual was ≤0.005 GPa. Elastic stiffness coefficients were derived by stress‐strain calculations.

## Conflict of interest

The authors declare no conflict of interest.

## Supporting information

As a service to our authors and readers, this journal provides supporting information supplied by the authors. Such materials are peer reviewed and may be re‐organized for online delivery, but are not copy‐edited or typeset. Technical support issues arising from supporting information (other than missing files) should be addressed to the authors.

Supplementary
