# Supplementary material for: Synthesis, Crystal Structure, and Compressibilities of Mn3−xIr5B2+x (0≤x≤0.5) and Mn2IrB2
Source: Chemistry. 2018 Sep 20;24(55):14679–85. doi: 10.1002/chem.201803235 (PMC6175196; doi:10.1002/chem.201803235)
Supplement: Supplementary file 1 — Supplementary [file CHEM-24-14679-s001.pdf]

CHEMISTRY
A European Journal

Supporting Information

Synthesis, Crystal Structure, and Compressibilities of $Mn_{3-x}Ir_5B_{2+x}$ ($0 \leq x \leq 0.5$) and Mn_2IrB_2

Benedikt Petermüller,[a] Christopher Neun,[b] Michal Stekiel,[b] Dominik Zimmer,[b] Martina Tribus,[c] Klaus Wurst,[a] Björn Winkler,[b] and Hubert Huppertz*[a]

chem_201803235_sm_miscellaneous_information.pdf

Supporting Information

Table S1: Atomic coordinates and equivalent anisotropic displacement parameters U_{eq} (Å^2) of Mn_2IrB_2.

Table S2: Interatomic distances (Å) in Mn_2IrB_2 derived from the single-crystal data.

Table S3: Atomic coordinates and isotropic displacement parameters U_{iso} (Å^2) of $Mn_{3-x}IrB_{2+x}$ ($0 \leq x \leq 0.5$).

Table S4: Interatomic distances (Å) in $Mn_{3-x}Ir_5B_{2+x}$ ($0 \leq x \leq 0.5$) derived from the powder crystal data.

Table S5: Pressure dependence of the unit cell parameters for $Mn_{3-x}Ir_5B_{2+x}$ ($0 \leq x \leq 0.5$).

Table S6: Pressure dependence of the unit cell parameters for Mn_2IrB_2.

Figure S1: Rietveld plot of $Mn_{3-x}Ir_5B_{2+x}$ ($0 \leq x \leq 0.5$).

Figure S2: Experimental powder pattern of Mn_2IrB_2.

Figure S3: Unit cell of Mn_2IrB_2 with the BM_6 units aligned in two different orientations. Boron atoms in red, manganese atoms in purple, and iridium atoms displayed in cyan. Atoms are shown with anisotropic displacement ellipsoids (probability: 90 %).

Figure S4: Unit cell of $Mn_{3-x}Ir_5B_{2+x}$ ($0 \leq x \leq 0.5$). Boron atoms in red, manganese atoms in purple, and iridium atoms displayed in cyan. Atoms are shown with anisotropic displacement ellipsoids (probability: 90 %).

Table S1: Atomic coordinates and equivalent anisotropic displacement parameters U_{eq} (Å^2) of Mn_2IrB_2.

atom	Wyckoff site	*x*	*y*	*z*	$U_{eq}{}^a$	s.o.f
Ir1	8*f*	1	0.56185(2)	0.09223(2)	0.0032(2)	1
Mn1	4*c*	0.5	0.5053(2)	0.25	0.0027(2)	1
Mn2/Ir2	8*f*	0.5	0.33538(8)	0.44150(6)	0.0035(2)	0.93/0.07(2)
Mn3	4*c*	0.5	0.2367(2)	0.25	0.0017(2)	1
B1	8*f*	0	0.3683(6)	0.3183(5)	0.0040(9)	1
B2	8*f*	0.5	0.6981(6)	0.1298(5)	0.0043(9)	1

$^a U_{eq}$ is defined as one third of the trace of the orthogonalized U_{ij} tensor

Table S2: Interatomic distances (Å) in Mn_2IrB_2 derived from the single-crystal data.

atom 1	atom 2	amount	distance (Å)	atom 1	atom 2	amount	distance (Å)
Ir1	B1	1 ×	2.244(6)	Mn1	B1	4 ×	2.257(5)
	B2	2 ×	2.123(4)		B2	2 ×	2.478(6)
	Mn1	2 ×	2.668(1)		Mn3	3 ×	2.648(2) - 2.767(2)
	Mn2	5 ×	2.731(2) - 2.733(1)		Ir1	4 ×	2.668(1)
	Mn3	1 ×	2.705(1)	Mn2	B2	3 ×	2.275(5) - 2.511(7)
	Ir1	1 ×	2.726(1)		B1	2 ×	2.283(5)
B1	B1	1 ×	1.808(2)		Mn3	1 ×	2.712(1)
	B2	1 ×	1.813(9)		Mn2	2 ×	2.771(2)
	Mn1	2 ×	2.257(5)		Ir1	5 ×	2.730(1) - 2.764(1)
	Mn2	2 ×	2.283(5)	Mn3	B1	4 ×	2.227(4)
	Mn3	2 ×	2.227(4)		B2	4 ×	2.264(5)
	Ir1	1 ×	2.244(6)		Mn1	3 ×	2.648(2) - 2.767(2)
B2	B1	1 ×	1.813(9)		Mn2	2 ×	2.712(1)
	Mn1	1 ×	2.478(6)		Ir1	2 ×	2.705(2)
	Mn2	3 ×	2.275(5) – 2.511(7)				
	Mn3	2 ×	2.264(5)				
	Ir1	2 ×	2.123(4)				

Table S3: Atomic coordinates and isotropic displacement parameters U_{iso} (Å^2) of $Mn_{3-x}Ir_5B_{2+x}$ ($0 \leq x \leq 0.5$).

atom	Wyckoff site	*x*	*y*	*z*	U_{iso}	s.o.f
Ir1	2*c*	0	0.5	0.5	0.0047(4)	1
Ir2	8*j*	0.2096(5)	0.6977(6)	0.5	0.0043(3)	1
Mn1	2*a*	0	0	0	0.005(3)	0.55(5)
B1	2*a*	0	0	0	0.005(3)	0.45(5)
Mn2	4*g*	0.3239(3)	0.8239(3)	0	0.0048(8)	1
B2	4*g*	0.1253(2)	0.6253(2)	0	0.006(5)	1

Table S4: Interatomic distances (Å) in $Mn_{3-x}Ir_5B_{2+x}$ ($0 \leq x \leq 0.5$) derived from the single-crystal data.

atom 1	atom 2	amount	distance (Å)	atom 1	atom 2	amount	distance (Å)
Ir1	B2	4 ×	2.18(1)	Mn1/B1	Mn1/B1	2 ×	2.882(1)
	Mn2	4 ×	2.724(2)		Ir2	8 ×	2.506(1)
	Ir2	4 ×	2.773(1)	Mn2	B2	3 ×	2.60(2) – 2.83(2)
	Ir1	2 ×	2.882(1)		Mn2	2 ×	2.882(1)
Ir2	B2	2 ×	2.16(1)		Ir1	2 ×	2.724(2)
	Mn1/B1	2 ×	2.506(1)		Ir2	8 ×	2.782(2) – 2.900(3)
	Mn2	4 ×	2.782(2) – 2.900(3)	B2	B2	2 ×	2.882(1)
	Ir1	1 ×	2.773(1)		Mn2	3 ×	2.60(2) – 2.83(2)
	Ir2	5 ×	2.882(1) – 2.900(1)		Ir1	2 ×	2.18(1)
					Ir2	4 ×	2.16(1)

Table S5: Pressure dependence of the unit cell parameters for $Mn_{3-x}Ir_5B_{2+x}$ ($0 \leq x \leq 0.5$).

p [GPa]	*a* [Å]	*c* [Å]	*V* [Å^3]
0	9.2850(2)	2.8823(5)	248.48(5)
3.3(1)	9.233(1)	2.865(1)	244.3(2)
6.4(2)	9.198(1)	2.865(1)	241.7(2)
9.4(3)	9.174(1)	2.857(1)	239.9(2)
13.1(4)	9.139(1)	2.850(1)	237.2(2)
16.8(5)	9.106(1)	2.825(1)	234.2(2)
19.8(6)	9.079(1)	2.816(1)	232.1(2)
23.7(7)	9.058(1)	2.801(1)	229.8(2)
27.9(9)	9.026(1)	2.782(1)	226.6(2)
31.3(1.0)	9.001(1)	2.773(1)	224.7(2)
36.6(1.1)	8.988(1)	2.750(2)	222.7(5)

Table S6: Pressure dependence of the unit cell parameters for Mn_2IrB_2.

p [GPa]	*a* [Å]	*b* [Å]	*c* [Å]	*V* [Å^3]
0	2.812(1)	10.091(1)	13.334(1)	378.5(3)
10	2.777(1)	9.988(1)	13.231(1)	366.9(3)
20	2.747(1)	9.896(1)	13.138(1)	357.1(3)
30	2.720(1)	9.816(1)	13.052(1)	348.5(3)
40	2.697(1)	9.745(1)	12.971(1)	340.9(3)
50	2.676(2)	9.680(1)	12.896(1)	334.1(3)

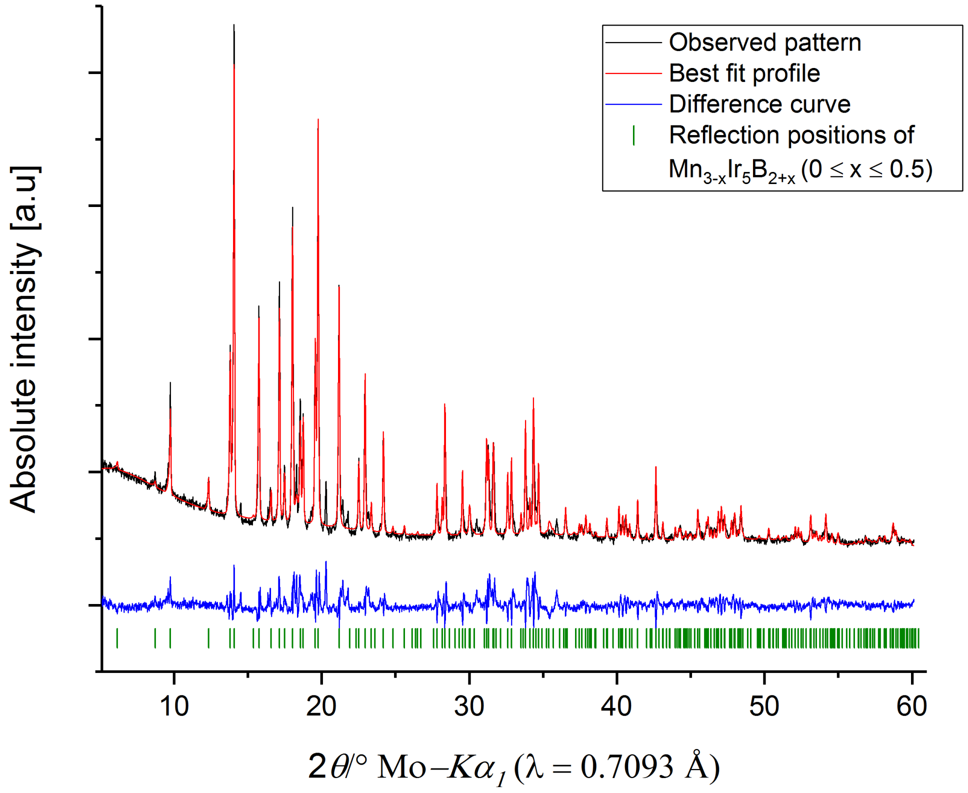

Figure S1: Rietveld plot of $Mn_{3-x}Ir_5B_{2+x}$ (0 ≤ x ≤ 0.5). Experimental powder diffraction pattern is shown in black, calculated pattern in red, and difference plot is shown in blue. The reflection positions of $Mn_{2.55}Ir_5B_{2.45}$ are shown in green.

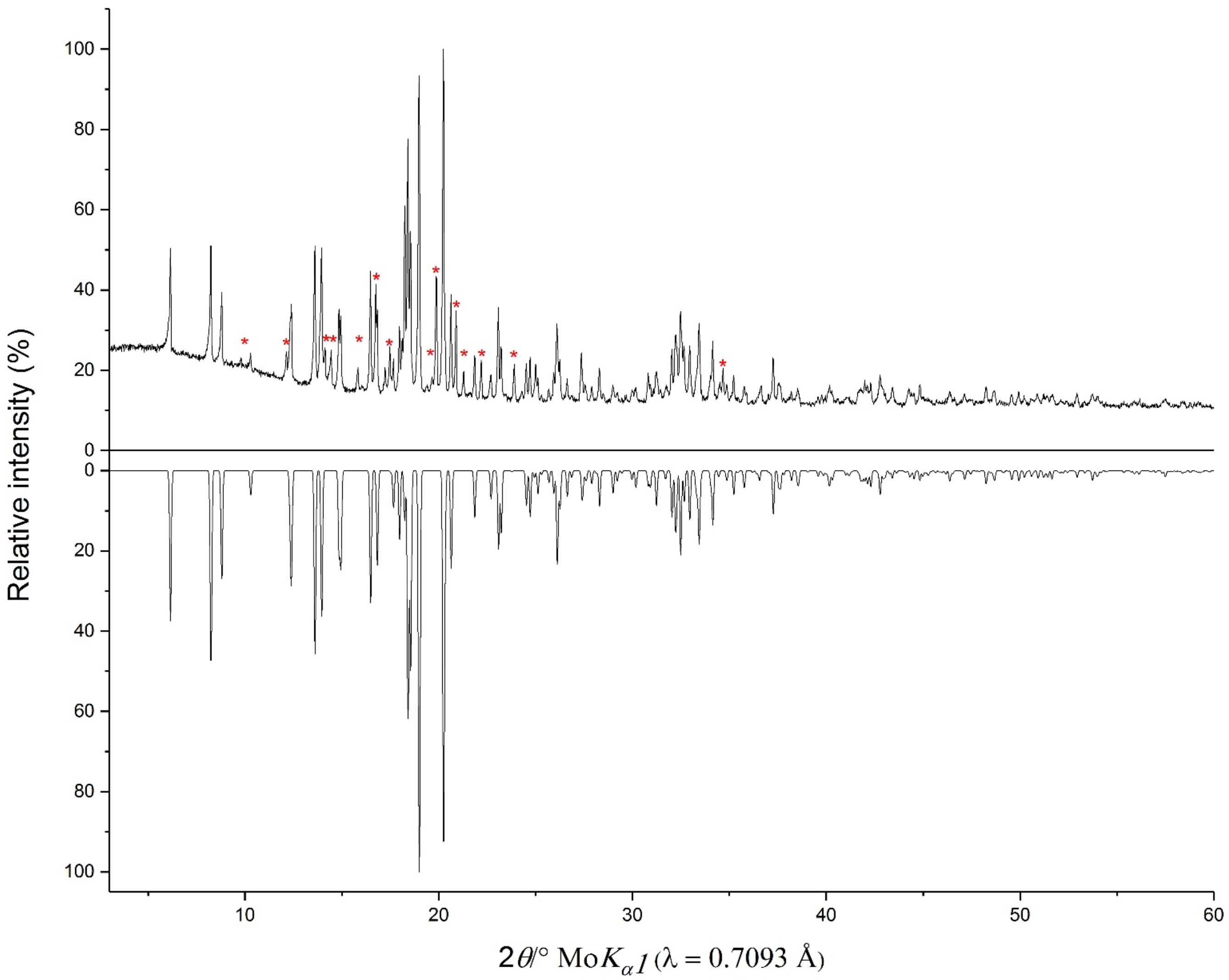

Figure S2: Top: experimental powder pattern of Mn_2IrB_2. The reflections marked with a red asterisk could not be assigned. Bottom: theoretical powder pattern of Mn_2IrB_2 based on single-crystal diffraction data.

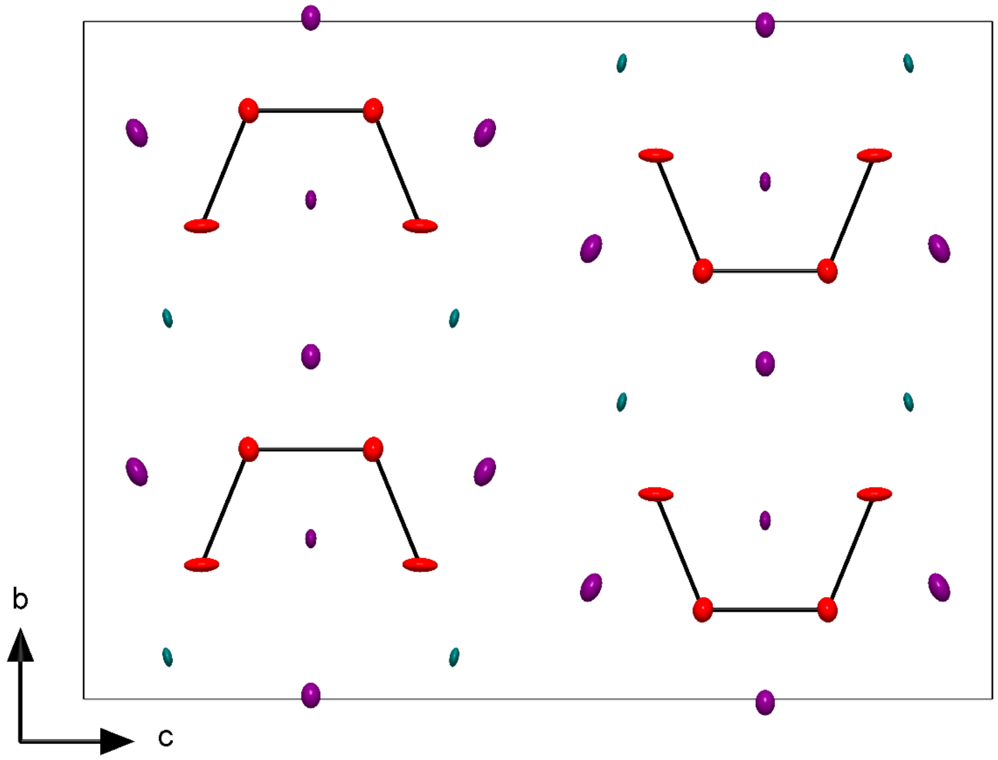

Figure S3: Unit cell of Mn_2IrB_2 with the BM_6 units aligned in two different orientations. Boron atoms in red, manganese atoms in purple, and iridium atoms displayed in cyan. Atoms are shown with anisotropic displacement ellipsoids (probability: 90 %).

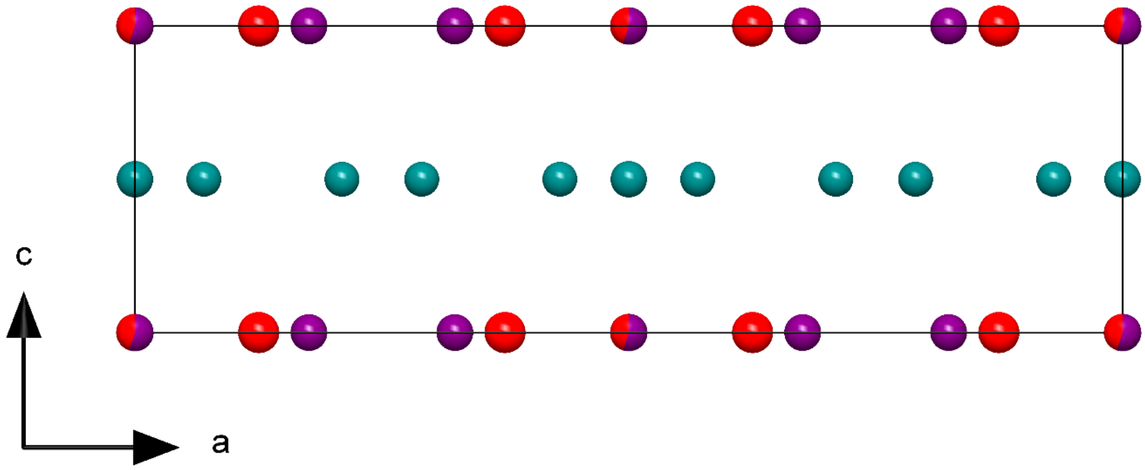

Figure S4: Unit cell of $Mn_{3-x}Ir_5B_{2+x}$ ($0 \leq x \leq 0.5$). Boron atoms in red, manganese atoms in purple, and iridium atoms displayed in cyan. Atoms are shown with anisotropic displacement ellipsoids (probability: 90 %).
